# Effect of Farm Management Practices on Morbidity and Antibiotic Usage on Calf Rearing Farms

**DOI:** 10.3390/antibiotics11020270

**Published:** 2022-02-18

**Authors:** Atte Sandelin, Outi Hälli, Heidi Härtel, Tuomas Herva, Liisa Kaartinen, Erja Tuunainen, Helena Rautala, Timo Soveri, Heli Simojoki

**Affiliations:** 1 Department of Production Animal Medicine, Faculty of Veterinary Medicine, University of Helsinki, Paroninkuja 20, FI-04920 Saarentaus, Finland; outi.halli@helsinki.fi (O.H.); helena.rautala@helsinki.fi (H.R.); timo.soveri@helsinki.fi (T.S.); heli.simojoki@helsinki.fi (H.S.); 2 HKScan Finland Ltd., Lemminkäisenkatu 48, FI-20520 Turku, Finland; heidi.hartel@hkscan.com; 3 Atria Ltd., Atriantie 1, FI-60550 Nurmo, Finland; tuomas.herva@atria.com; 4 Finnish Food Authority, Mustialankatu 3, FI-00790 Helsinki, Finland; liisa.kaartinen@ruokavirasto.fi; 5 Animal Health ETT, Huhtalantie 2, FI-60100 Seinäjoki, Finland; erja.tuunainen@ett.fi; 6 Department of Agricultural Sciences, Faculty of Agriculture and Forestry, University of Helsinki, Koetilantie 5, FI-00790 Helsinki, Finland

**Keywords:** morbidity, antibiotics, bovine respiratory disease, calf rearing farm

## Abstract

Antimicrobial resistance has been recognized as one of the top health threats to human society. Abundant use of antibiotics in both humans and animals has led to ever-increasing antibiotic resistance in bacteria. In food production, decreasing morbidity in beef herds would be an effective way to reduce the use of antibiotics. The objective of this retrospective observational study was to determine overall morbidity on calf rearing farms and to identify associated risk factors. Data were collected by questionnaire, meat companies’ databases and the national cattle register for 28,228 calves transported to 87 calf rearing farms. All medications given to these calves were retrospectively followed for 180 days from calf arrival to the farm. In total, 34,532 parenteral antibiotic medications were administered to the 28,228 study calves (122.3%), and 17,180 calves (60.9%) were medicated with antibiotics at least once during the follow-up. Higher numbers of calves transported to the same farm and larger age variation in calves in the same arrival batch were both associated with increased morbidity. In contrast, higher arrival age of individual calves was associated with decreased morbidity. Our study identifies several factors to consider in decreasing morbidity and antibiotic usage on calf rearing farms.

## 1. Introduction

High morbidity from infectious diseases and following intensive use of antibiotics has become a serious problem on calf rearing farms and in the beef production sector overall [1,2,3]. With the continued emergence of antibiotic-resistant bacteria, the attention of consumers and other stakeholders is directed to consumption of antibiotics in food production animals [4,5]. An association between antibiotic usage in cattle herds and the presence of antibiotic resistance was shown earlier [6]. Human infections caused by livestock-associated antibiotic-resistant bacteria (methicillin-resistant Staphylococcus aureus) have also been reported [7]. This development has driven the food producing industry to seek new methods to reduce antibiotic consumption. The best way to improve sustainable antimicrobial usage without causing animal welfare problems is to identify preventive methods and management practices to decrease the morbidity of food production animals.

In beef production, calf rearing farms are dependent on the calf supply of dairy farms, and many dairy farms are willing to sell their calves as early as possible. To ensure the supply of calves, it is common practice to transport calves to the calf rearing farms from multiple origin farms when calves are less than one month old [8,9,10]. To ensure social contacts between calves, enhancing animal welfare, major Finnish meat companies deliver calves only to group housing, although the legislation of the European Union allows individual housing for calves under 8 weeks of age [11]. These environmental and management-related factors on calf rearing farms facilitate the spread of infectious diseases between young, immunologically susceptible calves [12,13,14]. In addition, transportation and commingling of calves are major stress factors that further predispose calves to infections by weakening immunological responses [15,16]. Several studies have reported bovine respiratory disease (BRD) as the most common cause of increased treatment incidence on calf rearing farms [2,12]. Evaluating factors associated with calf morbidity enables the development of new practices to reduce morbidity and use of antibiotics on calf rearing farms. In addition, lower morbidity leads to more profitable beef production via better animal welfare, enhanced daily gain and decreased mortality [8].

Due to multifactorial etiology of the most common diseases, such as BRD and calf diarrhoea, extensive data are required to demonstrate the roles of different factors underlying increased morbidity [9,17,18]. Although several studies have reported factors associated with increased treatment incidence, more research is needed to determine the causal relationships [3,13,19,20,21,22]. Earlier studies have been conducted mainly in veal calf operations, which differ to some extent from Finnish calf rearing farms. In Finland, the vast majority of calves used for beef production are reared until the age of 18–20 months before slaughter. These calves are usually dairy breed bull calves or crossbred bull or heifer calves born on dairy farms. Approximately two-thirds of these calves are transported to the specialized calf rearing farms at the age of 10–30 days, where they are reared 4–6 months before transportation to the finishing farms. The remaining one-third of calves are transported directly to the fattening farms either before weaning (fattening farms for milk calves) or after weaning (fattening farms for weaned calves), where they are reared until slaughter.

Sick animals on Finnish calf rearing farms are medicated by either the veterinarian or farmer. Oral group treatments are not used on Finnish calf rearing farms, and metaphylactic group treatments are relatively uncommon; thus, the treatment incidence likely reflects the overall morbidity quite accurately. Since the revision of legislation in 2014, farmers may have medicines stored at the farm for medication of diseases that the veterinarian has diagnosed to occur commonly on the farm. The medication is carried out according to the farm’s health care veterinarian’s detailed instructions and under the veterinarian’s surveillance. No earlier studies have reported overall treatment incidences on Finnish calf rearing farms or the antimicrobial agents used on these farms. 

The primary objective of this study was to determine the overall treatment incidence of calves on Finnish calf rearing farms and to identify the factors associated with the treatment incidence. Our secondary objective was to determine the distribution of antimicrobial agents used on these farms. 

## 2. Results

### 2.1. Descriptive Statistics

Average follow-up time of the calves was 150.8 days (SD 27.8, range 0–180). Average time from arrival to first medication was 23.9 days (SD 25.9, range 0–173), varying between calf rearing farm types, being 22.9 days (SD 24.3, range 0–166) on specialized calf rearing farms, 35.3 days (SD 38.4, range 0–173) on fattening farms for milk calves and 35.5 days (SD 25.7, range 8–103) on fattening farms for weaned calves. Consumption of antibiotics varied considerably between farm types, being on average 1.22 treatments per calf during follow up (SD 1.39, range 0–11). Treatment incidence was highest on specialized calf rearing farms and lowest on fattening farms for weaned calves (Table 1). Recurrent medications were common and 9299 (53.3%) of all medicated calves were treated more than once during follow-up. Of all administered antibiotic treatments, 9854 (28.5%) were given as the only medicine and 24,678 (71.5%) were given in combination with an NSAID. NSAIDs were given as only medicine 2681 times (7.2% of all given medications). Most of the study farms (*n* = 68; 79.1%) had certain criteria decided in advance by the farm’s health care veterinarian to guide which calves should be treated with antibiotics, NSAIDs or both. Descriptive statistics are presented in Table 2, Table 3, Table 4 and Table 5. 

Average sizes of the calf rearing farms varied considerably between different farm types and meat companies. Average farm size was largest for specialized calf rearing farms (661 heads), followed by fattening farms for milk calves (497 heads) and weaned calves (85 heads). Average size of the calf rearing farms contracted to meat company A was 682 heads, to meat company B 608 heads and to meat company C 239 heads. Size of calf rearing farms was highly correlated with number of calves transported to these farms (0.87). An average calf batch consisted of calves from 27 origin farms (SD 21, range 1–181). The average number of origin farms per calf batch on specialized calf rearing farms was 32, on fattening farms for milk calves 19, and on fattening farms for weaned calves five. 

On the average farm, 35.7% (SD 30.8, range 0–99.7%) of the calves were medicated at least once with antibiotics during the follow-up. Similarly, when recurring treatments were taken into account, the medication proportion was 56.9% (SD 65.0, range 0–309.0%). The corresponding proportions for different farm types were 48.5% (SD 30.8, range 3.8–99.7%) and 83.0% (SD 74.3, range 3.7–309.0%) for specialized calf rearing farms, 30.7% (SD 24.8, range 0–87.1%) and 40.9% (SD 40.0, range 0–151.4%) for fattening farms for milk calves, and 4.7% (SD 11.5, range 0–40.9%) and 5.0% (SD 11.8, range 0–40.9%) for fattening farms for weaned calves, respectively. Only 13 farms out of 87 (14.9%) did not have any antibiotic treatments during the study period. Of these 13 farms, 11 were fattening farms for weaned calves and two were fattening farms for milk calves. Only one farm (1.2%) reported the use of regular metaphylactic antibiotic treatments for controlling diseases, and 22 farms (25.3%) reported the use of metaphylactic group treatments only if necessary. 

**Figure 1:**
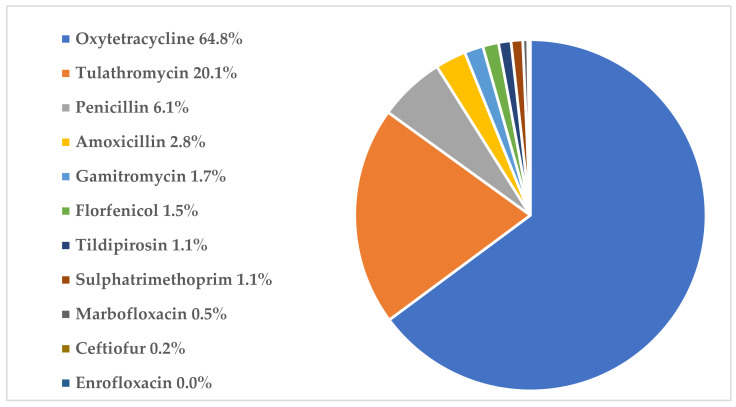
Proportions of antibiotic treatments administered during follow-up according to the active substance. All treatments were given individually to the calves using parenteral administration (topical treatments excluded, *n* = 132, 0.4% of all antibiotic treatments).

During the follow-up, no oral antibiotic treatments were recorded. Study calves received in total 34,553 individual parenteral antibiotic medications (topical treatment excluded, *n* = 132). Oxytetracycline was the most commonly used active substance with 22,405 recorded courses, followed by tulathromycin with 6955 recorded courses. Detailed proportions of active substances used for treatments during the follow-up are presented in Figure 1. BRD was the most common treatment indication (89.7% of registered indications, *n* = 30 256). Proportions of the other indications were 4.0% for interdigital phlegmon (*n* = 1347), 1.6% for umbilical inflammation (*n* = 551), 1.4% for gastrointestinal diseases (*n* = 486) and 3.3% for all other combined rarer indications (*n* = 1099). Finnish legislation requires farmers to take samples for laboratory identification of causative pathogens and to determine the antibiotic susceptibility profile of bacteria, if the same disease signs are repeatedly treated with antibiotics or if group treatment with antibiotics is required. However, only 22 farms (25.3%) declared taking samples from sick animals annually. Twenty-eight farms (32.2%) and 37 farms (42.5%) took samples for laboratory testing at random intervals or not at all, respectively. 

Biosecurity issues were considered to varying degrees on study farms. Continuous filling of milk-feeding compartments was rare compared with emptying the compartments between calf batches (13.7% vs. 86.3%). More than half (54.8%) of the farms washed and disinfected milk-feeding compartments between calf batches, whereas one-third (34.2%) only washed the compartments, and the remainder (11%) only mechanically cleaned or occasionally cleaned the compartments between batches. Most farms did not group arriving calves into separate pens according to any specific criteria. The most common criterion was health status of the calf, which was used as a grouping criterion by 12 farms (13.8%). 

### 2.2. Predictors Associated with a Calf’s Odds to Become Medicated

During the follow-up, 17,435 calves (61.8%) were medicated at least once with antibiotics or NSAIDs or both. Results of the mixed multivariate logistic regression model are presented in Table 6. An increased number of calves transported to the farm and higher age variation in arriving calf batches were associated with a calf’s higher odds of being medicated (OR 1.141, *p*-value 0.002 and OR 1.090, *p*-value 0.007, respectively). Calves reared on farms with natural ventilation in compartments for weaned calves had significantly lower odds to become medicated during follow-up than calves on farms with mechanical ventilation (OR 0.393, *p*-value 0.028). Ventilation type of the weaned calf barn was also highly correlated with the insulation of the barn (chi-squared test, *p*-value < 0.001). Of 66 mechanically ventilated barns, 65 (98.5%) were also insulated, whereas only seven (41.2%) of 17 naturally ventilated barns were insulated. Predictably, metaphylactic use of antibiotics was associated with a higher proportion of medicated calves. On the other hand, calves with a higher arrival age had significantly lower odds to be medicated during the follow-up (OR 0.981, *p*-value < 0.001). Other factors associated with decreased odds were more caretakers for every 100 calves, and heifer calf and medicines not stored at the farm. Moreover, Holstein, crossbred Aberdeen Angus, crossbred Limousine and crossbred Blonde d’Aquitaine breeds were associated with lower odds to being medicated relative to Ayrshire calves. Intraclass correlation was low at both farm level (0.249) and arrival batch level (0.453).

### 2.3. Predictors Associated with Calf’s Possibility to Become Repeatedly Medicated

Of all study calves, 32.9% were medicated more than once during the follow-up. Numbers of calves medicated at least once, and calves medicated recurrently during the follow-up are presented in more detail in Table 7. 

The multivariate mixed Poisson regression model included several factors associated with the risk of recurrent medications (Table 8). A higher number of calves transported to the farm was associated with increased incidence risk of recurrent medications (IRR 1.023, *p*-value < 0.001). In the case of multiple milk-feeding compartments, filling up the incoming calves to each compartment one by one with a shorter filling-up interval increased the incidence risk compared with simultaneous filling of calves to all compartments with a longer filling-up interval. In addition, leaving calves to rear in the milk-feeding compartment after weaning increased the incidence risk. On the other hand, higher arrival age and not measuring temperature to detect sick calves decreased the incidence risk of recurrent medications. Holstein calves, crossbred Blonde d’Aquitaine calves and calves from other breeds were associated with a decreased incidence risk relative to Ayrshire calves.

## 3. Discussion

### 3.1. Use of Antibiotics on Calf Rearing Farms

Antibiotic consumption was highest on specialized calf rearing farms, followed by fattening farms for milk calves and weaned calves. One factor explaining this finding could be the relatively larger number of young cattle on specialized calf rearing farms, where most cattle are younger than six months. These farms also buy more cattle during the year than farms rearing the calves until slaughter age, which was associated with increased treatment incidence risk in the current and earlier studies [23,24]. In total, 33.9% of all calves reared on specialized calf rearing farms did not receive any antibiotic treatment during the follow-up, but recurrent treatments were common in those that were treated. In the current study, calves were usually medicated with a combination of antibiotics and NSAIDs (71.5%) rather than an antibiotic alone (28.5%). Recommendations to combine NSAIDs with antibiotic treatment are based mostly on empirical evidence, although data on the benefits of this combination to treat BRD are not solid [25,26,27]. Use of different antibiotics to treat common infections in study calves adhered to the treatment recommendations of the Finnish food authority [28]. In these recommendations, oxytetracycline is the primary treatment option, and tulathromycin a second choice for BRD. Tulathromycin is especially used on farms where *Mycoplasma bovis* has been diagnosed in BRD cases. Oxytetracycline and tulathromycin products comprised 84.9% of given antibiotics, and BRD was the indication for medication in 89.7% of the treatments. Our results support earlier findings of BRD being the predominant cause of morbidity in calf rearing facilities [2,12,21]. 

### 3.2. Calf Level Factors Associated with Medication Incidence on Calf Rearing Farms

Statistical models constructed to analyze the data collected from calf rearing farms revealed several factors associated with treatment incidence on these farms. A calf’s arrival age, sex and breed were all associated with the calf’s probability of being medicated during the follow-up. Higher arrival age significantly decreased the odds of being medicated during follow-up, and also decreased the incidence risk of recurrent medications. According to our knowledge, no earlier study has reported association between arrival age of the calf and treatment incidence on a calf rearing farm. Goetz et al. [22] presented in their recent study the decreasing effect of higher arrival weight on morbidity in a veal calf facility. The immune system of young calves develops gradually during the first months of life [14]. The more mature immune system of older calves might protect them from infectious diseases. Older and heavier calves might also have an advantage in competition for resources on calf rearing farms. Compared with the Ayrshire breed, Holstein, crossbred Aberdeen Angus, crossbred Limousine and crossbred Blonde d’Aquitaine calves all had lower odds of being medicated during follow-up. Holstein calves and crossbred Blonde d’Aquitaine calves also had a decreased incidence risk for recurrent medications. To the authors’ knowledge, a similar breed effect has not been reported in earlier studies. In our registry data, breed of the calf was registered according to the breed of the calf’s sire. This means that we do not know the breed of the dam, and it is not taken into account in the analysis. In Finland, practically all calves reared on calf rearing farms have a dairy farm origin, and these calves are very rarely pure beef breed, whereas cross breeding of milk breeds on dairy farms is a rather uncommon practice in Finland.

Higher variation in age of calves arriving in the same batch was associated with higher odds of being medicated at least once during follow-up. According to the authors’ knowledge, similar findings have not been reported in earlier studies. Age variation can be increased by adding older calves to the calf batch that were rejected from the primary transport due to sickness and treated already at the dairy farm. They could carry infection to younger susceptible calves on the calf rearing farm. Higher variation in calves’ ages in the same batch might also lead to an uneven competition situation between younger and older calves, causing stress to younger calves. Stress is known to affect calves’ immunity in several ways and predispose calves to BRD [16,29]. Lava et al. [19] reported an association between high weight differences in calves of the same group (>100 kg) and increased mortality. More studies are needed to confirm all contributing factors behind this phenomenon. In the present study, grouping calves at the time of arrival to their own pens according to the calf’s age or weight did not significantly affect medication probability. Recurrent medications may also reflect susceptibility of some individuals to diseases or poor efficacy of the medications used to treat these conditions. Moreover, a bout of illness might predispose the individual to other pathogens and diseases [30].

### 3.3. Farm Level Factors Associated with Medication Incidence on Calf Rearing Farms

Larger number of calves transported to the farm during follow-up was associated with the calf’s higher odds of being medicated at least once during the follow-up and also increased incidence risk of recurrent medications. Commingling cattle from different origin farms has been shown to increase morbidity in earlier studies [24,31]. Probably, more origin farms lead to a higher number of different pathogens presented to the herd, thus increasing the morbidity of the calves [20,24]. It also increases the risk of simultaneous presence of potentially infective calves and susceptible newly arrived calves at the same farm. In our data, the number of calves transported to the calf rearing farms was correlated with both herd size and number of different origin farms among calves transported to the farm. An association between larger herd size and higher calf mortality has been demonstrated in previously published reports [23,32]. Contract with meat company C was associated with increased risk of the calf of being medicated at least once, or even recurrently, during the follow-up compared with meat company A. We have to be cautious with this finding, because of unbalance in geographical locations of the contract farms of different meat companies. Meat company C has farms in the most intensive cattle production area in Finland, which might affect prevalence of BRD by increased infection pressure. Geographical location may also affect the management and other policies on the farms. Meat companies also directly affect their contract farms in many ways, including different advisory services and organizations for animal transportations. Bokma et al. [3] reported a similar association between veal calf companies and antimicrobial usage on their contract veal calf farms. 

In Finland, the legislation allows veterinarians to equip farmers who have a cattle healthcare contract with the veterinarian with certain antibiotics and other medicines. In this contract, the farmers are obligated to maintain medical book-keeping in a web-based national herd health register (Naseva), and they are only allowed to use the medicines for indications as outlined by the contract veterinarian. In addition, farmers are obligated to have regular health care visits by the contract herd health veterinarian. In the present study, calves reared on farms that did not have medicines available on the premises had lower odds of being medicated during the rearing period. It could be that farms having problems with increased morbidity are more prone to have a contract with a veterinarian and medicines available on the farm. One possible explanation could be that farmers who have medicines on the premises have a lower threshold to use them to treat sick calves. However, it is also possible that some calves are more easily left untreated on farms that do not store medicines.

Our results also showed that calves reared on farms with less caretakers in relation to the number of calves had significantly higher odds of being medicated during follow-up. The number of caretakers on the farm affects the time available for overall management. If time is too limited, this leads to less attention to preventing infectious diseases. We also observed that calves reared on farms where temperature was not measured to detect sick calves had significantly lower odds of being medicated recurrently. These phenomena might be explained by several different factors. Farms that do not measure temperature might also have a higher threshold for medicating sick calves, or the number of sick calves may be underestimated because without temperature measurement part of the sick calves may go undetected.

Natural ventilation in the barn for weaned calves was associated with the calves’ lower odds of being medicated during the follow-up compared with barns with mechanical ventilation. A similar finding was made in the studies by Brscic et al. [13] and Schnyder et al. [20]. Brscic et al. [13] noted that veal calves reared in mechanically ventilated barns had more observable nasal discharge two weeks before slaughter than calves reared in naturally ventilated barns, although no such difference was reported in the fattening period (week 3 and 13 after arrival to the farm). Schnyder et al. [20] observed that treatment incidence increased in mechanically ventilated barns compared with naturally ventilated barns. Increased odds for medications in mechanically ventilated barns could partly be explained by potentially smaller air spaces in these barns and formation of draft due to mechanically enhanced airflow. The inside temperature in the barn can potentially vary between naturally and mechanically ventilated barns. According to our data, insulated barns were significantly more often mechanically ventilated (94.4%) than uninsulated barns (0%). Naturally ventilated barns are also usually built with a larger air space to enable natural air ventilation. 

Associations between some factors measuring internal biosecurity and treatment incidence were examined in our study. In the case of multiple milk-feeding compartments, simultaneous filling of incoming calves to all compartments with a longer filling-up interval decreased the risk for recurrent medications compared with filling up calves to compartments one by one with a shorter filling-up interval. A possible explanation for this finding could be that when calves arrive at the farm continuously there are more occasions of stressed and potentially infection-spreading calves on the farm. These calves potentially spread infections to older calves that have already adapted to living in the new environment. In addition, leaving calves in the same compartment after weaning from milk instead of moving them to another compartment increases the risk of recurrent medications. This phenomenon is difficult to explain. Possibly, the compartments where calves were reared during the milk-feeding period and after weaning are difficult to design to be optimal for both age groups. Older calves need more floor space and more air space to ensure good hygiene in the compartment. Proper cleaning and disinfection of the compartments are not usually done before those are empty from animals. However, the questionnaire data in our study did not specify how long calves were kept in the same compartment after weaning. Lava et al. [19] reported higher treatment incidences in calves kept in the same air space with other groups of calves. In our study, defining whether compartments on some farms are categorized as their own separate air space or not was particularly difficult. Some farms have compartments with their own air ventilation for each arriving calf batch, but the feeding trough is in the same air space with other compartments, or alternatively there is a door between two separate compartments. Here, a separate air space was defined as space with no direct air connection with other compartments. In our previous study [8], based on the same data as the present study, we reported higher mortality rates in calves reared on farms with continuous filling of milk-feeding compartments, but no similar result was detected for treatment incidence here.

Correlation between recurrent medications and both calf mortality and daily gain on calf rearing farms was reported in a recent study based on the same data as used in our study [8]. In that study, higher morbidity was associated with increased mortality such that calves medicated more than two separate times had significantly higher odds of suffering untimely death than calves not medicated. Given medications during the rearing period also linearly reduced the daily gain of calves starting from the first treatment and decreasing the daily gain gradually if recurrent medications were given. According to these findings, decreasing treatment incidence via decreasing morbidity on calf rearing farms would not only decrease the use of antibiotics in beef production but also have a significant economic and animal welfare-enhancing effect. More studies are needed to thoroughly understand the complex relationship between incidence of BRD, treatment rate and mortality.

### 3.4. Study Population

Our stratified study sample was randomly selected among the contract farms of the three biggest meat companies in Finland. The vast majority of Finnish beef producers are contracted with one of these three meat companies. During the data collection, 68 out of 155 farms were excluded. Most of the exclusions occurred because of missing or unavailable medication records (*n* = 54), which might be one potential source of selection bias. Only seven farms out of 155 disclosed their unwillingness to hand over their production data for study purposes. However, farms keeping a book on medications according to legal requirements, and willing to put in extra effort, are probably better represented in our sample population. Farmers keen to maintain accurate bookkeeping might be also more conscientious with other farm practices. On the other hand, due to legislation, farmers are obligated to maintain medical bookkeeping in electronic form in the national cattle herd health register (Naseva) if they want to store and administer medicines on the farm. This might lead to a bias where larger farms with more health problems and greater need for daily medications are over-represented in the sample. 

During the data collection, calves were retrospectively assigned to calf batches according to their arrival date and questionnaire results. This assignment was not always straightforward, at least not for the farms with continuous filling (i.e., receiving calves continuously). Nevertheless, only 5.6% of the calves were reared on farms with continuous filling of milk-feeding compartments. Due to the overall strict antibiotic use policy on Finnish calf rearing farms, the given treatments describe rather accurately the actual morbidity on farms. In many other studies where antibiotic group treatments are used in a prophylactic or metaphylactic manner, a similar relationship cannot be assumed based on the amounts of antibiotic treatments or daily doses administered. In treatment calculations for this study, we calculated treatments as separate cases if time between treatments was at least seven days. So, we believe the treatments describe quite well separate courses. However, we cannot be completely sure if recurrently treated calves were treated for the relapse or reinfection. To reliably distinguish these two possible outcomes, more accurate microbiological data and molecular genetic methods would be needed for pathogenic comparison. In the current study, season of the year when calves were transported to the farm was not found to have significant effect on studied outcomes. However, our data included only calves transported to the calf rearing farms during the nine months period, which is not enough to cover all seasons of the year. In Finland, four seasons of the year differ greatly from each other, and results could have been different if all four seasons would have been present in the data. 

Our study included many kinds of farms, and there can be difference in main predisposing factors for BRD between farm types. Our study design was able to find major factors affecting treatment rates, but more focused studies are needed to find practical solution to lower treatment rates on specialized large scale calf rearing farms with highest treatment rates. Finland is free from some of the major pathogens common in the largest veal producing countries, including, for example, infectious bovine rhinotracheitis and bovine viral diarrhea. Despite this, we consider that most of the results of this study can be extrapolated to calf rearing farms also in other countries.

## 4. Materials and Methods

The present study was part of a larger project that was conducted to determine production parameters on Finnish calf rearing farms (daily weight gain of calves, use of medicines, calf morbidity and mortality) and to identify methods to positively affect these outcomes (for more details, see Sandelin et al. [8]).

### 4.1. Study Population and Study Design

This retrospective observational study was designed to determine overall treatment incidence on Finnish calf rearing farms and to specify associated factors. In addition, medicine usage on Finnish calf rearing farms was determined. The original study sample consisted of 155 calf rearing farms contracted with the three largest meat companies in Finland. The numbers of different farm types (specialized calf rearing farms, fattening farms for milk calves, fattening farms for weaned calves) were weighted according to their actual prevalence among the contract farms. As inclusion criteria, farms must have received a minimum of 50 calves during the nine-month study period (between 1 January 2016 and 1 October 2016) and have continued production without major changes in calf management practices or farm facilities during this time. Study farms were randomly selected among the contract farms, and the final stratified sample included 65 specialized calf rearing farms, 60 fattening farms buying milk calves and 30 fattening farms buying weaned calves. The numbers of contract farms were evenly divided between meat companies. If the number of farms for one meat company was too small to fill the needed sample size, the missing farms were compensated by adding more same type farms from other meat companies. A lottery conducted by a member of the study group was used to randomize the selection process. 

A letter was sent to all included farms, and farmers were asked to contact their meat company in case of unwillingness to disclose their production data for study purposes. At this point, seven farmers refused to participate, and one farm was excluded because the calves received were older than 150 days. The remaining farmers were telephoned and submitted a questionnaire by the project veterinarian. In this questionnaire, availability of the medicine bookkeeping of the farm was inquired. In total, 54 farms were excluded due to lack of accurate medical bookkeeping data. In addition, six farms were excluded due to missing registry data (*n* = 2) or questionnaire data (*n* = 4). In all, 68 farms were excluded, and the final study sample consisted of 87 calf rearing farms.

During the study period 28,687 calves were transported to the study farms. In total, 459 calves were excluded from the study. Most of these calves (*n* = 365) were excluded because of exceptionally high transportation age (>60 days when transported to the specialized calf rearing farm or fattening farm for milk calves or >179 days when transported to the fattening farm for weaned calves). Five calf rearing farms had an additional dairy herd or suckler cattle herd included under the same herd ID. All calves born in these suckler herds and heifer calves born in dairy herds were excluded from the study (*n* = 66). In addition, 19 calves were excluded due to unexplainable short rearing period (<60 days), eight calves due to unexplainable transportation age (0 days) and one calf due to inaccurate registry data. The final study population comprised 28,228 calves. Most of the included calves were transported to the 45 specialized calf rearing farms (*n* = 23,946), the second most to the 28 fattening farms for milk calves (*n* = 3746) and the third most to the 14 fattening farms for weaned calves (*n* = 536). Medications used were retrospectively followed for 180 days starting from the day of arrival at the farm. Arrival date and questionnaire data were used to assign calves to arrival batches.

### 4.2. Data Collection

Individual data on calves involved in the study, as well as data on dairy herds of origin, were collected from the national cattle register. Individual calf data included sex, breed, date of birth, date of arrival and departure from study farm and possible date and cause of death. Breed of the calf was registered according to the breed of the sire. The calf’s origin dairy farm data included the size of the origin farm (average number of animals on the farm in 2016) and calf mortality on the origin dairy farm (from birth to six months of age, stillborns included). 

Overall, data of calf management and medication practices followed on the farm were inquired in a questionnaire that was presented by telephone to the responsible person in the farm. The questionnaire consisted of 49 closed questions divided into four topics: general management on the farm; management of calves in milk feeding; management of calves after weaning, and policies related to treatments and medications. Answering all questions took 15–30 minutes. As part of the validation process, the questionnaire was proofread by several experts (herd health veterinarians and a farm management adviser) and piloted with five calf rearing farms by comparing the results of the questionnaire with the actual practices on the farms. In the questionnaire, and in this study context, milk-feeding compartments refer to the sections of the barn that are separated from each other with walls. In these compartments, calves are kept either in a single pen or several separate pens. Size of the calf rearing farm (maximum herd size) in the year 2016 was inquired as part of the questionnaire.

All antibiotic and nonsteroidal anti-inflammatory drug (NSAID) medications given during the first 180 days of the rearing period were collected for the analysis. If a calf died or was further transported earlier, the follow-up time was shorter. For 66 farms, medication data were available in electronic form in the national cattle herd health register (Naseva). For an additional 21 farms, medication data were available in paper form. These records were collected via email, mail or by visiting the farm. All the collected medication records included at least the EU identification number of the medicated calf, the date of medication and the medicine used. In addition, most of the records also included the indication for medication, amount of medicine used, length of the course and identification of the medicine giver. Medications were given and registered by either the veterinarian or the farmer according to the veterinarian’s instructions. Medications given for dehorning were deleted, and other collected medication data were combined with each calf’s individual data. In addition, the number of antibiotic courses and the number of overall medications given to each calf were calculated. Antibiotic treatments (including all routes of administration) were counted as separate courses if there was at least seven days between the medication events or the antibiotic was changed to another active substance. Similarly, overall medications given to each calf were counted such that medication events were treated as separate if time between medications was more than seven days regardless of the medicine used. In calculations to determine the distribution of different parenterally given antibiotic active substances (topical treatments excluded), and medication events were calculated as separate if there was more than two days between given treatments.

### 4.3. Statistical Analysis

The sample size was calculated such that it allowed us to detect at least a 1% difference in a calf’s probability of being medicated at least once during the rearing period and of being medicated repeatedly with a power of 0.9. Clustering within herds (estimated intra-class correlation (ICC) 0.1) was taken into account and significance level was set at 0.05. All statistical analyses were performed in Stata/MP 14.1 for Windows (StataCorp LP, TX, USA).

Outcome variables for the statistical analysis were a calf’s possible medical treatment at least once during the follow-up (binomial variable, medicated 0/1) and the risk of a calf being recurrently medicated during the follow-up (count variable, number of medications) (Table 7). To clarify the results, the number of recurrent medications was categorized as presented in Table 7. The individual calf was used as an experimental unit in both generated models. Univariate analysis was conducted for 29 independent variables for both outcome variables (Table 2, Table 3, Table 4 and Table 5). Mixed logistic regression model for “medicated 0/1” and mixed Poisson regression model for “recurrent medications” were conducted for the univariate analysis. Both the arrival batch and the calf rearing farm were used as grouping variables in both univariate analyses. A predictor variable was selected to the construction of the multivariate model if an association with the outcome variable was detected (*p* < 0.2). If a correlation between two independent variables was detected (correlation coefficient > 0.6), only the biologically more relevant variable was retained in the model. To clarify the results, the number of calves transported to the calf rearing farm and the size of the calf’s origin dairy herd were divided by 100. In addition, the total number of calves reared in milk-feeding compartments and size of the calf groups in milk-feeding compartments were categorized into four categories in the questionnaire (Table 3). Breeds were categorized such that rarer breeds, including Brown Swiss, crossbred Hereford, crossbred Charolais, crossbred Simmental, Highland cattle, Jersey, Montbéliard and rural Finnish cattle, were combined under the name “other breed”. The correlations between herd size, number of calves transported to the farm and number of different origin dairy farms of arriving calves were evaluated using Spearman rank correlation. 

A multivariate mixed logistic regression model and a multivariate mixed Poisson regression model were generated to determine factors associated with outcome variables “medicated 0/1” and “number of medications”, respectively. Only calves with at least one medication event during the rearing period were included to the multivariate mixed Poisson regression model. Both models were constructed using a stepwise-backwards method by excluding the nonsignificant factors. After the inclusion–exclusion process, 12 and 8 independent variables were preserved in the final models, respectively. Meat company, farm and arrival batch-level clustering were taken into account. Rearing farm and calf’s arrival batch were used as random variable in both models. Meat company and farm type were used as fixed effects in both models. The confounding effect was controlled by forcing breed of the calf into both models. The independent variables calf’s weight and age at time of arrival were highly correlated, and due to better usability in practice, the age of the calf was preserved in the model. In both models, significance level was set at *p* < 0.05. None of the biologically significant interactions seemed to have a marked effect. The basic assumption for a logistic model (linear relationship between log odds of outcome and continuous variable) was inspected. A logistic regression model fit was assessed visually by plotting the predicted successes against the observed successes. No serious breaches of the underlying assumptions were detected. Furthermore, the area under the ROC curve (0.9) was evaluated to assess predictive ability of the logistic model. A Poisson model fit was assessed by computing deviance and Anscombe residuals. Both residuals seemed to be approximately normally distributed.

In total, 27,824 calves were included in the multivariate mixed logistic regression model for a calf’s probability of being medicated at least once during the follow-up. Missing calves were excluded due to either missing complete questionnaire data (*n* = 402) or missing data of arrival batch (*n* = 2). In the multivariate mixed Poisson regression model for recurrent medications during the rearing period, 16,435 calves were included and 11,793 calves excluded from the model. Of the excluded calves, 10,793 were excluded due to zero medications during the study period and the rest due to missing data on overall management on the farm (*n* = 1000).

## 5. Conclusions

We report high individual medication rates of calves on Finnish calf rearing farms, even though oral antibiotic treatments and group treatments are absent or very rarely utilized. Higher age of the calf at time of arrival to the farm was a protective factor against increased treatment incidence. In contrast, high variation in arrival ages of calves in the same arrival batch increased the risk of a calf to be medicated at least once during follow-up. Our results give us a reason to find out ways to increase and equalize transportation ages of calves on a national level. Increased profitability, better animal welfare and lower antibiotic usage in beef production can be achieved by successful reduction of morbidity on calf rearing farms. 

## Figures and Tables

**Table 1:** Number of separate medications given to study calves (*n* = 28,228) during the 180-day follow-up.

		Total *n* = 28,228	Specialized Calf Rearing Farm *n* = 23,946	Fattening Farm for Milk Calves *n* = 3746	Fattening Farm for Weaned Calves *n* = 536
		(*n*/%)	(*n*/%)	(*n*/%)	(*n*/%)
**Number of calves medicated at least once during follow-up**	**Antibiotic**	17,180/60.9	15,820/66.1	1331/35.5	29/5.4
	Antibiotic, NSAID or both *	17,435/61.8	16,010/66.9	1391/37.1	34/6.3
**Number of medications when recurrent medications are taken into account**	Antibiotic **	34,532/122.3	32,721/136.6	1780/47.5	31/5.8
	Antibiotic, NSAID or both ***	33,403/118.3	31,526/131.7	1841/49.2	36/6.7

* NSAID = Nonsteroidal anti-inflammatory drug; ** Number of medications calculated as separate if time between medications was more than 7 days or active substance of antibiotic treatment was changed. *** Number of medications calculated as separate if time between medications was more than 7 days.

**Table 2:** Descriptive statistics of calf and farm level data for 28,228 calves reared in 87 calf rearing farms and univariate associations of predictor and outcome variables.

Variable	Total Calves/Farms	% Calves/Farms	Medicated at Least Once (%)				Average Number of Medication Events per Calf			
			*n*	%	*p*-Value	Missing	*n*	Average *	*p*-Value	Missing
**Farm type**										
Specialized calf rearing farm	23,946/45	84.8/51.7	23,946	66.9	ref.	0	16,010	1.956	ref.	7936
Fattening farm for milk calves	3746/28	13.3/32.2	3746	37.1	0.010	0	1391	1.324	<0.001	2355
Fattening farm for weaned calves	536/14	1.9/16.1	536	6.3	<0.001	0	34	1.059	0.004	502
Sum:	28,228/87	100/100	28,228			0	17,435			10,793
Missing:	0/0									
Wald-test:					<0.001				<0.001	
**Contract meat company**										
Company A	18,304/40	64.8/46.0	18,304	66.7	ref.	0	12,033	1.848	ref.	6271
Company B	7819/39	27.7/44.8	7819	46.3	0.148	0	3622	2.089	<0.001	4197
Company C	2105/8	7.5/9.2	2105	84.6	0.002	0	1780	1.903	0.182	325
Sum:	28,228/87	100/100	28,228			0	17,435			10,793
Missing:	0/0									
Wald-test:					<0.001				<0.001	
**Farm receives additional older** animals **										
No	10,569/50	37.4/57.5	10,569	52.0	ref.	0	5501	2.012	ref.	5068
Yes	17,659/37	62.6/42.5	17,659	67.6	0.333	0	11,934	1.854	<0.001	5725
Sum:	28,228/87	100/100	28,228			0	17,435			10,793
Missing:	0/0									
**Calf mortality on origin dairy farm**										
0–2%	4463/-	15.8/-	4463	59.1	ref.	0	2639	1.868	ref.	1824
2.1–5.9%	8617/-	30.5/-	8617	60.4	0.612	0	5204	1.900	0.632	3413
6–9.9%	7435/-	26.3/-	7435	61.4	0.998	0	4567	1.896	0.207	2868
10% or more	7713/-	27.4/-	7713	65.1	0.017	0	5025	1.934	0.218	2688
Sum:	28,228/-	100/-	28,228				17,435			10,793
Missing:	0/-									
Wald-test:					0.004				0.487	
**Sex**										
Bull	25,018/-	88.6/-	25,018	61.6	ref.	0	15,403	1.919	ref.	9615
Heifer	3210/-	11.4/-	3210	63.3	<0.001	0	2032	1.787	0.398	1178
Sum:	28,228/-		28,228			0	17,435			10,793
Missing:	0/-									
**Calf breed**										
Finnish Ayrshire	10,818/-	38.3/-	10,818	62.2	ref.	0	6733	1.967	ref.	4085
Holstein	11,405/-	40.4/-	11,405	61.9	<0.001	0	7060	1.907	<0.001	4345
Aberdeen Angus	1111/-	3.9/-	1111	57.2	0.202	0	635	1.865	0.126	476
Limousine	1331/-	4.7/-	1331	61.4	<0.001	0	817	1.846	0.435	514
Blonde d’aquitaine	2563/-	9.1/-	2563	61.6	<0.001	0	1579	1.764	<0.001	984
Other breeds	1000/-	3.5/-	1000	61.1	<0.001	0	611	1.653	<0.001	389
Sum:	28,228/-	100/-	28,228			0	17,435			10,793
Missing:	0/-									
Wald-test:					<0.001				<0.001	

* Average number of separate medication events for one calf during the follow-up when medicated at least once during the rearing period (scale: one to five or more medication events). ** Calves transported ≥ 60 days of age to specialized calf rearing farms and fattening farms for milk calves or ≥180 days of age to the fattening farms for weaned calves.

**Table 3:** Descriptive statistics of management and compartmentation data for 28,228 calves reared in calf rearing farms and univariate associations of predictor and outcome variables. Milk-feeding variables are only available for specialized calf rearing farms and fattening farms for milk calves.

Variable	Total Calves/Farms	% Calves/Farms	Medicated at Least Once (%)				Average Number of Medication Events per Calf			
			*n*	%	*p*-Value	Missing	*n*	Average *	*p*-Value	Missing
**Number of calves in one milk-feeding compartment**										
1–20 calves	1014/11	3.6/15.1	1014	25.2	ref.	0	256	1.316	ref.	758
21–40 calves	6272/28	22.7/38.3	6272	37.3	0.177	0	2367	1.500	0.006	3905
41–80 calves	17,098/27	61.7/37.0	17,098	75.9	<0.001	0	12,972	2.072	<0.001	4126
81–100 calves	3308/7	12.0/9.6	3308	54.6	0.046	0	1806	1.327	0.266	1502
Sum:	27,692/73	100/100	27,692			0	17,401			10,291
Missing:	536/14									
Wald-test:					<0.001				<0.001	
**Sizes of the calf groups in milk-feeding compartments**										
1–10 calves	516/4	1.9/5.5	516	24.2	ref.	0	125	0.332	ref.	391
11–20 caves	4437/25	16.0/34.2	4437	46.3	0.661	0	2054	0.566	0.193	2383
21–30 calves	9660/21	34.9/28.8	9660	73.6	0.123	0	7111	1.584	0.012	2549
More than 30 calves	13,079/23	47.2/31.5	13,079	62.0	0.140	0	8111	0.724	0.018	4968
Sum:	27,692/73	100/100	27,692			0	17,401			10,291
Missing:	536/14									
Wald-test:					0.068				<0.001	
**Milk-feeding compartments operated as all in/all out**										
Yes	26,145/63	94.4/86.3	26,145	64.0	ref.	0	16,741	1.925	ref.	9404
No	1547/10	5.6/13.7	1547	42.7	0.677	0	660	1.403	0.011	887
Sum:	27,692/73	100/100	27,692	6.3		0	17,401			10,291
Missing:	536/14									
**Arriving calves are grouped to the pens according to**										
Body weight	4545/12	17.7/16.9	4545	46.0	ref.	0	2092	1.461	ref.	2453
Health status	639/2	2.5/2.8	639	41.5	0.877	0	265	1.381	0.669	374
Age	1411/4	5.5/5.7	1411	36.7	0.647	0	518	1.241	0.237	893
Calves kept in same groups as during transportation	564/2	2.2/2.8	564	73.4	0.051	0	414	1.019	0.412	150
Random	18,107/48	70.6/67.6	18,107	66.4	0.696	0	12,022	1.915	<0.001	6085
Some other criteria	398/3	1.5/4.2	398	39.5	0.657	0	157	1.573	0.588	241
Sum:	25,664/71	100/100	25,664			0				
Missing:	2564/16									
Wald-test:					0.425				<0.001	
**Filling of compartments for arriving milk calves**										
All compartments filled simultaneously	10,203/46	37.5/66.7	10,203	50.6	ref.	0	5160	1.828	ref.	5043
All compartments filled independently	17,015/23	62.5/33.3	17,015	71.2	0.004	0	12,108	1.944	<0.001	4907
Sum:	27,218/69	100/100	27,218			0	17,268			9950
Missing:	1010/18									
**Washing and disinfection of milk-feeding compartments between calf batches**										
Washing and disinfection	17,093/40	61.7/54.8	17,093	65.8	ref.	0	11,247	2.058	ref.	5846
Only washing	9402/25	34.0/34.2	9402	60.1	0.848	0	5647	1.642	0.107	3755
Only mechanical cleaning	1161/7	4.2/9.6	1161	43.4	0.626	0	504	1.470	0.021	657
Only occasional cleaning	36/1	0.1/1.4	36	8.3	0.284	0	3	1.333	0.544	33
Sum:	27,692/73	100/100	27,692				17,401			10,291
Missing:	536/14									
Wald-test:					0.721				<0.044	
**Air connection between compartments**										
Milk-feeding compartment has own air space	10,630/43	38.9/59.7	10,630	37.4	ref.	0	3978	1.320	ref.	6652
Air connection between milk-feeding and other compartments	16,700/29	61.1/40.3	16,700	78.2	0.001	0	13,062	2.109	<0.001	3638
Sum:	27,330/72	100/100	27,330			0	17,040			10,290
Missing:	898/15									
**Handling of calves after weaning**										
Calves relocated to new compartment	19,647/54	78.5/79.4	19,647	58.5	ref.	0	11,503	1.848	ref.	8144
Calves stay in the same compartment	6679/14	21.5/20.6	6679	74.3	0.007	0	4965	2.040	<0.001	1714
Sum:	26,326/68	100/100	26,326	50.8		0	16,468			8144
Missing:	1902/19									
**Air ventilation in compartments for weaned calves**										
Mechanical ventilation	22,768/66	81.8/76.7	22,768	68.2	ref.	0	15,522	1.936	ref.	7246
Natural ventilation	4401/17	15.8/19.8	4401	29.3	0.057	0	1288	1.392	0.002	3113
Combination of mechanical and natural ventilation	657/3	2.4/3.5	657	37.4	0.824	0	246	1.398	0.155	411
Sum:	27,826/86	100/100	27,826				17,056			10,770
Missing:	402/1									
Wald-test:					0.151				0.003	
**Air ventilation in compartments for weaned calves**										
Mechanical ventilation	22,768/66	81.8/76.7	22,768	68.2	ref.	0	15,522	1.936	ref.	7246
Natural ventilation	4401/17	15.8/19.8	4401	29.3	0.057	0	1288	1.392	0.002	3113
Combination of mechanical and natural ventilation	657/3	2.4/3.5	657	37.4	0.824	0	246	1.398	0.155	411
Sum:	27,826/86	100/100	27,826				17,056			10,770
Missing:	402/1									
Wald-test:					0.151				0.003	
**Compartmentation of weaned calves**										
Weaned calves in own air space	8351/32	30.2/37.7	8351	39.8	ref.	0	3327	1.283	ref.	5024
Weaned calves in same compartment and air space with older cattle	6126/42	22.1/49.4	6126	35.0	0.239	0	2143	1.380	<0.001	3983
Weaned calves in own compartment but same air space with older cattle	13,206/11	47.7/12.9	13,206	87.7	<0.001	0	11,579	2.156	<0.001	1627
Sum:	27,683/85	100/100	27,683			0	17,049			10,634
Missing:	545/3									
Wald-test:					<0.001				<0.001	

* Average number of separate medication events for one calf during the follow-up when medicated at least once during the rearing period (scale: one to five or more medication events).

**Table 4:** Descriptive statistics of medication practices followed in 87 calf rearing farms with 28,228 reared calves and univariate associations of predictor and outcome variables.

Variable	Total Calves/Farms	% Calves/Farms	Medicated at Least Once (%)				Average Number of Medication Events per Calf			
			*n*	%	*p*-Value	Missing	*n*	Average *	*p*-Value	Missing
**Temperature measured to detect sick calves**										
Yes	20,416/54	73.4/62.1	20,416	68.9	ref.	0	14,058	2.038	ref.	6358
No	7812/33	26.6/37.9	7812	43.2	0.008	0	3377	1.348	<0.001	4435
Sum:	28,228/87	100/100	28,228			0	17,435			10,793
Missing:	0/0									
**Temperature measured systemically from all calves in the same group if at risk of illness**										
Yes	3151/11	11.2/12.6	3151	66.6	ref.	0	2097	1.923	ref.	1054
No	25,077/76	88.8/87.4	25,077	61.2	0.193	0	15,339	1.901	<.0.001	9738
Sum:	28,228/87	100/100	28,228			0	17,436			10,792
Missing:	0/0									
**Medicines stored at farm for future use**										
Yes	26,850/66	95.1/75.9	26,850	64.1	ref.	0	17,215	1.914	ref.	9635
No	1378/21	4.9/24.1	1378	16.0	<0.001	0	220	1.127	<0.001	1158
Sum:	28,228/87	100/100	28,228			0	17,435			10,793
Missing:	0/0									
**Medication policy on farm**										
Only sick animals medicated	19,676/64	69.7/73.6	19,676	60.2	ref.	0	11,809	1.756	ref.	7867
Metaphylactic group treatments used if needed	8552/23	30.3/26.4	8552	65.8	0.001	0	5626	1.974	<0.001	2926
Sum:	28,228/87	100/100	28,228			0	17,435			10,793
Missing:	0/0									
Use of vaccination against BRD **										
Yes	1293/4	4.6/4.6	1293	56.1	ref.	0	726	1.332	ref.	567
No	26,935/83	95.4/95.4	26,935	62.0	0.990	0	16,709	1.929	0.371	10,226
Sum:	28,228/87	100/100	28,228			0	17,435			10,793
Missing:	0/0									

* Average number of separate medication events for one calf during the follow-up when medicated at least once during the rearing period (scale: one to five or more medication events). ** Bovilis Bovipast RSP parenteral vaccination was used on every farm. Vaccine includes following inactivated pathogens: Bovine respiratory syncytial -virus, Parainfluenza-3-virus and *Mannheimia haemolytica.*

**Table 5:** Descriptive statistics of continuous variables of 28,228 calves and univariate associations of predictor and outcome variables. Study included 23,946 calves reared in 45 specialized calf rearing farms, 3746 calves reared on 28 fattening farms for milk calves and 536 calves reared in 14 fattening farms for weaned calves. Daily gain was calculated for calves with two weight measurements.

	*n* Calves/Farms	Average Calf/Farm level	SD Calf/Farm level	Medicated 0/1				Number of Medication Events per Calf			
				*n*	OR	*p*-Value	Missing	*n*	IRR	*p*-Value	Missing
**Herd size**	28,228/87	628/289	446/296	28,228	1.004	<0.001	0	17,435	1.000	<0.001	10,793
Missing:	-/-										
**Herd size of the origin dairy farm**	28,226/87	122/117	104/-	28,226	1.001	<0.001	0	17,434	1.000	0.550	10,794
Missing:	2/-										
**Number of calves transported to farm**	28,228/	956/324	722/455	28,228	1.002	<0.001	0	17,435	1.030	<0.001	10,793
Missing:	0 /0										
**Arrival age of the calf/days**				28,228	0.979	<0.001	0	17,435	0.996	<0.001	10,793
Specialized calf rearing farm	23,946/45	23/22.3	9.2/3.7								
Fattening farm for milk calves	37,46/28	21.5/21.0	8.7/3.6								
Fattening farm for weaned calves	536/14	97.4/97.6	33.2/23.8								
Total:	28,228/87	24.2/34.0	14.4/29.7								
Missing:	-/-										
**Arrival weight of the calf/kg**				27,724	0.985	<0.001	504	17,221	0.998	0.001	10,503
Specialized calf rearing farm	23,536/45	58.5/58.6	10.1/2.6								
Fattening farm for milk calves	3659/28	57.5/57.3	9.4/2.9								
Fattening farm for weaned calves	529 (14)	117.4/117.9	39.4/21.3								
Total:	27,724/87	59.5/67.7	14.1/23.9								
Missing:	504/0										
**Average arrival age of calves in the same batch**				28,228	0.955	<0.001	0	17,435	<1.000	0.874	10,793
Specialized calf rearing farm	23,946/45	23.0/22.3	5.3/3.7								
Fattening farm for milk calves	3746/28	21.5/21.0	5.1/3.6								
Fattening farm for weaned calves	536/14	97.4/97.6	24.0/23.8								
Total:	28,228/87	24.2/34.0	11.9/29.7								
Missing:	0/0										
**Age variation in arrival batch (SD)**				28,226	0.986	0.608	2	17,434	1.004	0.401	10,793
Specialized calf rearing farm	23,946/45	7.4/7.2	1.8/1.7								
Fattening farm for milk calves	37,46/28	6.7/6.6	2.4/2.1								
Fattening farm for weaned calves	534/14	22.4/21.8	9.1/7.0								
Total:	28,224/87	7.6/9.3	3.1/6.3								
Missing:	4/0										
**Number of caretakers/100 calves**				28,228	1.541	<0.001	0	17,435	0.725	<0.001	10,793
Specialized calf rearing farm	23,946/45	0.6/1.0	0.4/0.6								
Fattening farm for milk calves	3746/28	0.7/0.8	0.4/0.5								
Fattening farm for weaned calves	536/14	1.6/1.6	0.6/0.6								
Total:	28,228/87	0.6/1.00	0.4/0.6								
Missing:	0										

**Table 6:** Mixed multivariate logistic regression model to study the association between a calf’s odds being medicated at least once during the maximum 180-day follow-up, and predictor variables in 87 herds. Average follow-up time for 28,228 study calves was 150.8 days when early transportation to the finishing units and untimely deaths are taken into account.

Predictor Variable	Odds Ratio	*p*-Value	95% Confidence Interval	
Number of calves transported to the farm/100 *	1.141	0.002	1.049–1.240	
**Age of calf on arrival**	0.981	<0.001	0.977–0.985	
**Arrival age variation in calf batch (standard deviation)**	1.090	0.007	1.024–1.160	
**Number of caretakers/100 calves**	0.294	<0.001	0.149–0.580	
**Farm type**				
Specialized calf rearing farm	ref.	ref.		
Fattening farm for milk calves	0.569	0.179	0.250–1.296	
Fattening farm for weaned calves	0.212	0.123	0.029–1.524	
Wald-test:		0.181		
**Contract meat company**				
Meat company A	ref.	ref.		
Meat company B	0.876	0.717	0.427–1.796	
Meat company C	15.208	<0.001	4.749–48.700	
Wald-test:		<0.001		
**Sex**				
Bull	ref.	ref.		
Heifer	0.723	<0.001	0.642–0.814	
**Breed**				
Ayrshire	ref.	ref.		
Holstein	0.807	<0.001	0.747–0.873	
Crossbred Aberdeen Angus	0.628	<0.001	0.526–0.750	
Crossbred Limousine	0.797	0.007	0.675–0.940	
Crossbred Blonde d’aquitaine	0.746	<0.001	0.654–0.851	
Other breeds **	0.951	0.593	0.790–1.145	
Wald-test:		<0.001		
**Type of ventilation for weaned calves**				
Mechanical ventilation	ref.	ref.		
Natural ventilation	0.393	0.028	0.171–0.905	
Partly mechanical and natural ventilation	0.918	0.919	0.176–4.780	
Wald-test:		0.088		
**Medicines stored at farm for future use**				
Yes	ref.	ref.		
No	0.131	<0.001	0.045–0.387	
**Medication policy on farm**				
Metaphylactic group treatments used if needed	ref.	ref.		
Only sick animals medicated	0.307	0.001	0.152–0.620	
**Temperature measured to detect sick calves**				
Yes	ref.	ref.		
No	0.534	0.031	0.268–1.067	

* Number of calves transported to the farm divided by 100. ** Brown swiss, Hereford, Charolais, Simmental, Highland cattle, Jersey, Montbéliard and Finnish cattle.

**Table 7:** Descriptive statistics of outcome variables (medicated 0/1 and number of medications) of 28,228 calves on 87 farms. The study included 23,946 calves reared on 45 specialized calf rearing farms, 3746 calves reared on 28 integrated beef production farms and 536 calves reared on 14 finishing farms for weaned calves.

	Total	%	%
	Number of Calves	Proportion of All Calves	Average at Farm Level
**Medicated at least once during follow-up**			
Yes	17,435	61.8	36.6
No	10,793	38.2	63.4
Total:	28,228	100	100
Missing:	0		
**Number of medication events during follow-up**			
Zero	10,793	38.2	
One	8158	28.9	
Two	5036	17.9	
Three	2565	9.1	
Four	1110	3.9	
Five or more	566	2.0	
Total:	28,228	100.0	
Missing:	0/0	0/0	

**Table 8:** Multivariate mixed Poisson regression model to study the association between a calf’s incidence rate ratio (IRR) of being recurrently medicated during the 180-day follow-up and predictor variables in 87 herds. The average follow-up time for 28,228 study calves was 150.8 days when early transportations to the finishing units and untimely deaths were taken into account.

Predictor Variable	IRR	*p*-Value	95% Confidence Interval
Number of calves transported to the farm/100 *	1.023	<0.001	1.020–1.026
**Age of calf on arrival**	0.997	<0.001	0.995–0.998
**Age variation in arrival batch (standard deviation)**	1.010	0.091	0.998–1.021
**Contract meat company**			
Meat company A	ref.	ref.	
Meat company B	0.971	0.684	0.850–1.121
Meat company C	1.320	0.007	1.080–1.615
Wald-test:		0.008	
**Breed**			
Ayrshire	ref.	ref.	
Holstein	0.949	<0.001	0.926–0.973
Crossbred Aberdeen Angus	0.954	0.129	0.897–1.014
Crossbred Limousine	0.984	0.572	0.931–1.040
Crossbred Blonde d’Aquitaine	0.900	<0.001	0.863–0.939
Other breeds **	0.876	<0.001	0.820–0.937
Wald-test:		<0.001	
**Filling of compartments for arriving milk calves**			
All compartments filled simultaneously	ref.	ref.	
All compartments filled independently	1.221	0.002	1.073–1.388
**Handling of calves after weaning**			
Calves relocated to new compartment	ref.	ref.	
Calves stay in the same compartment	1.094	<0.001	1.052–1.136
**Temperature measured to detect sick calves**			
Yes	ref.	ref.	
No	0.779	< 0.001	0.743–0.817

* Number of calves transported to the farm divided by 100. ** Brown swiss, Hereford, Charolais, Simmental, Highland cattle, Jersey, Montbéliard and Finnish cattle.

## Data Availability

Not applicable.
